# Sex Stratified Treatment of Neurological Disorders: Challenges and Perspectives

**DOI:** 10.3390/brainsci10020103

**Published:** 2020-02-14

**Authors:** Ivan Nalvarte

**Affiliations:** Department of Biosciences and Nutrition, Karolinska Institutet, 141 57 Huddinge, Sweden; ivan.nalvarte@ki.se; Tel.: +46-8-52481148

**Keywords:** sex hormone, sex chromosome, gender, neurobiology, neurodegeneration

## Abstract

Despite the obvious sex differences in many of the most common neuropsychiatric and neurodegenerative disorders, males and females are still often treated the same from a clinical perspective. Why is that? The simple answer is that there is still too little known about this very complex matter. Sex hormone signaling, genetic sex, sex-biased comorbidities, and social gender perceptions all interact, making discrimination between the impacts of each of these factors hard to evaluate. Adding to the complexity is that important species differences must be taken into account when interpreting data from animal models. Clearly, to overcome this, larger efforts are needed that incorporate epidemiological, experimental, and clinical data to provide a solid scientific base for more personalized and informed clinical decisions that will benefit both men and women suffering from neurological disorders.

Sex differences are well known in many neurological disorders. Perhaps the most well-studied are the sex differences in depression and anxiety that are both more prevalent in females, and that show a good correlation with circulating gonadal hormone levels [1]. For instance, depression as a result of menopause or following child birth is well acknowledged in the clinic. However, despite increasing evidence showing sex differences in the development and/or progression of most neuropsychiatric and neurodegenerative disorders [2,3,4], biological sex is rarely taken into consideration when making clinical treatment decisions. A major obstacle behind this is the complexity of the issue. In addition to the still vast gap in mechanistic knowledge about the actions of sex hormones and sex chromosome genes in neurological disorders, gender-associated sociological factors, such as stereotypes, education, differences in latencies to seek clinical contact, as well as sex-biased comorbidities (e.g., cardiovascular disease), contribute to the complexity and thus to the lack of sex-stratified clinical recommendations or treatments. Large efforts are needed to overcome this. Several funding and regulatory bodies are taking measures to ensure the inclusion of both sexes in most animal research and in clinical trials. This is a welcome initial step in the right direction that must be accompanied by larger, long-term funded multidisciplinary initiatives that combine epidemiologic, experimental and clinical research to provide a solid scientific base for better informed clinical recommendations and decisions. Clearly, regulatory bodies must be involved in promoting such research.

The recent article by Pinares-Garcia and colleagues [3] reviews the latest knowledge about sex differences in neuropsychiatric and neurodegenerative disorders and discuss the sociocultural aspects that may, in part, influence the clinical manifestation and treatment of the disorders. They also illustrate the importance of understanding the different contributions of sex hormones and sex chromosome genes in neurodevelopment and in the adult brain in order to understand the impact of these factors on neurological disorders. Such knowledge has been much dependent on animal experiments, which have provided invaluable insights into the sexual differentiation of the brain and the impact of sex hormones on neuroprotection. However, great caution must be taken when translating animal data to human physiology. For instance, the organizational imprinting of sex specification in rodents is dependent on the conversion of testosterone to estradiol by aromatase during the masculinization of the developing male rodent brain [5,6]. The fetal rodent brain is also well-protected from high estradiol levels by efficient estradiol sequestering by alpha-fetoprotein [7]. In humans however, alpha-fetoprotein has a low affinity for estradiol [8,9]. Instead, the sex hormone binding globulin protein (SHBG) may have a similar function to the alpha-fetoprotein, except for that it has a higher affinity for androgens than estradiol [10]. This means that circulating sex hormones in humans may have different physiological effects than in rodents, and that SHBG in humans may protect the female brain from excess androgen levels and masculinization. Naturally, this must be taken into account when interpreting animal data.

Despite important species differences, animal studies using the 4-core genotype [11] and the sex chromosome trisomy [12] models have provided pivotal understanding about the different contributions of sex hormones and sex chromosome genes to human neurobiology. The recent advances in using human stem cells as neurological disease models holds great promise to further accelerate our mechanistic understanding behind the etiology of neurodevelopmental and neurodegenerative disorders [13,14]. A great advantage is that these cells can be obtained and reprogrammed from patients suffering from different neurological diseases. On top of this, the development and lineage commitment, as well as functionality of the cells, can be amenably followed, and since culture conditions are defined, exposure to sex hormones on either a male (XY) or female (XX) genetic background can easily be studied. Further development of human stem cell models to more complex 3D systems, such as brain organoids or assembloids, will be important tools for the study of human neurobiology [15,16,17]. Linking such experimental models to population-wide epidemiological data on, for example, hormone replacement therapy use or genome-wide association studies will be of paramount importance to advance our understanding of the sex-differences in neurological disorders.

As Pinares-Garcia and colleagues point out [3], although gender-specific medicine is slowly getting recognition, challenges still exist when it comes to its full clinical implementation. To this end, a better understanding of the interactions between sex hormones, genetic sex, and sociological gender factors is needed. Such knowledge will be a base for better, more personalized, treatment strategies adapted to men and women suffering from neurological disorders.
